# Effects of a health worker-led 3-month yoga intervention on blood pressure of hypertensive patients: a randomised controlled multicentre trial in the primary care setting

**DOI:** 10.1186/s12889-021-10528-y

**Published:** 2021-03-20

**Authors:** Raja Ram Dhungana, Zeljko Pedisic, Suira Joshi, Mahesh Kumar Khanal, Om Prakash Kalauni, Anu Shakya, Vijay Bhurtel, Savyata Panthi, K. C. Ramesh Kumar, Binod Ghimire, Achyut Raj Pandey, Bihungum Bista, Shiva Ram Khatiwoda, Craig Steven McLachlan, Dinesh Neupane, Maximilian de Courten

**Affiliations:** 1 Institute for Health and Sport, Victoria University, Melbourne, Australia; 2 Ministry of Health, Kathmandu, Nepal; 3 Nepal Ayurveda Research and Training Center, Kathmandu, Nepal; 4 DFID/NHSP3/MEOR, Abt Associates, Kathmandu, Nepal; 5 Nepal Health Research Council, Kathmandu, Nepal; 6 Patanjali Ayurveda Medical College and Research Center, Dhulikhel, Nepal; 7 Health Faculty, Torrens University, Sydney, Australia; 8 Welch Center for Prevention, Epidemiology and Clinical Research, Department of Epidemiology, Johns Hopkins University, Baltimore, MD USA; 9 Nepal Development Society, Bharatpur, Chitwan Nepal; 10 Mitchell Institute for Education and Health Policy, Victoria University, Melbourne, Chitwan Australia

**Keywords:** Hypertension, Blood pressure, Primary care, Yoga, Ayurveda, Nepal

## Abstract

**Background:**

Hypertension control remains a major challenge globally. A recent systematic review suggested that yoga has beneficial effects on reducing blood pressure. However, the role of yoga in hypertension management in primary health care has received little attention, and no studies have evaluated the impact of a yoga program fully delivered by health care staff on hypertension. This study, therefore, assessed the effects of a health worker-led yoga intervention on blood pressure reduction among hypertensives patients in the primary care setting.

**Methods:**

This was a multicentric, two-arm, randomised trial conducted among hypertensive patients in seven Ayurveda Health Centres in Nepal between March 2017 and June 2018. One hundred and twenty-one participants who were on or without medications were randomised to intervention (*n* = 61) and wait-list control (*n* = 60) groups using stratified block randomisation. Participants in the intervention arm received an intervention consisting of an initial five-day structured yoga training at the centres and then a further home-based practice of yoga for five days a week for the following 90 days. Both intervention and control groups also participated in a 2-h health education session. The primary outcome of this trial was systolic blood pressure at 90-day follow-up. Data were analysed on an intention-to-treat basis using linear mixed-effects regression models.

**Results:**

We included all 121 study participants (intervention/control = 61/60) in the primary analysis (52.1% males; mean ± SD age = 47.8 ± 10.8 years). The difference in systolic blood pressure between the intervention group and the control group was − 7.66 mmHg (95% CI: − 10.4, − 4.93). For diastolic blood pressure, the difference was − 3.86 mmHg (95% CI: − 6.65, − 1.06). No adverse events were reported by the participants.

**Conclusions:**

A yoga program for hypertensive patients consisting of a five-day training in health centres and 90 days of practice at home is effective for reducing blood pressure. Significant benefits for hypertensive patients could be expected if such programmes would become a part of the standard treatment practice.

**Trial registration:**

This trial was prospectively registered with the Clinical Trial Registry of India [CTRI/2017/02/007822] on 10/02/2017.

**Supplementary Information:**

The online version contains supplementary material available at 10.1186/s12889-021-10528-y.

## Background

Hypertension is a major public health problem globally affecting around 22% of the world’s adult population in 2015 [1]. Together with the growing burden of hypertension, the prevalence of untreated and uncontrolled hypertensive is also very high, particularly in low-and middle- income countries (LMICs). Among all hypertensives in LMICs in 2010, 29.0% were treated, and only 7.7% had controlled blood pressure [2]. In Nepal, for example, more than half of those who are treated still have uncontrolled blood pressure [3–5].

Beyond medication, several non-pharmacological measures are available that can contribute to the effective management of hypertension. To reduce systolic blood pressure by 4–11 mmHg in hypertensive individuals, the new American College of Cardiology/American Heart Association guidelines recommend weight loss, “heart-healthy” diet, potassium supplementation, sodium reduction, increasing physical activity and limiting alcohol intake [6]. Using culturally accepted and evidence-based non-pharmacological measures alongside the medication may further achieve optimal control of high blood pressure in low resource primary care settings. Niu et al. [7] found that combining non-drug therapies with antihypertensive medications could further improve blood pressure reduction targets.

The use of yoga for controlling high blood pressure is an increasingly popular intervention [8–10]. It has shown positive effects not only on hypertension but also on a wide range of other health conditions [9, 11–14]. In the most recent systematic review of 49 clinical trials, Wu et al. [15] suggested that yoga is a viable antihypertensive lifestyle therapy. The findings showed that practising yoga at least three times a week is associated with a reduction in systolic blood pressure (SBP) and diastolic blood pressure (DBP) by 10 mmHg and 6 mmHg, respectively [15]. Despite a large number of clinical trials on the effects of yoga on hypertension, there are relatively few studies conducted in primary care settings [16, 17], and none of them has involved existing health workers in implementing yoga programs. If yoga is to be used as an adjunct or primary initial lifestyle therapy to control hypertension in clinical settings, primary care facility-based yoga training led by clinical staff could be a feasible approach.

Given that the studies conducted in a real-world setting are more likely to be translated into practice and to minimize the gap between the evidence and practice [18, 19], this study aimed to assess the effects of structured yoga practice on blood pressure reduction among hypertensive patients in primary healthcare facilities.

## Methods

### Trial design

This was a multicentric, two-arm, randomised, wait-list controlled, nonblinded trial comparing structured yoga practice (alongside health education) against health education only over three months. It was conducted among 121 hypertensive participants in seven Ayurveda Health centres (AHCs) in Nepal between March 2017 and June 2018. The study is reported using CONSORT and Intervention Description and Replication (TIDieR) guidelines.

### Study participants

Study participants were first-stage hypertensive patients attending outpatient departments at the trial centres, who had high blood pressure (SBP of ≥140 mmHg and < 160 mmHg or DBP of ≥85 mmHg and < 100 mmHg) or had been taking antihypertensive medication with SBP of ≥130 mmHg and < 160 mmHg or DBP of ≥85 mmHg and < 100 mmHg based on clinical measurements on two occasions, 1–2 weeks apart. The criteria for participant selection were age (≥18 years and ≤ 70 years) irrespective of gender and medication history. Persons with diabetes, those with a known case of secondary hypertension and/or other cardiovascular diseases/conditions, pregnant women, and those who practised yoga for 30 days or more in the previous 6 months were excluded. The hypertensive patients at each AHC were screened for eligibility criteria. Once the patients agreed to enrol in the study by providing their written informed consent, their de-identified codes and study sites were shared to the statistician (recruited outside the author team) who performed randomisation. The statistician did not have access to any other data about the participants. We used the centrally generated stratified block randomisation list to allocate the participants in the intervention and wait-list control groups, with recruiting site as a stratifying variable. A total of 121 hypertensive participants were recruited and allocated to intervention (*n* = 61) and control (*n* = 60) groups using the above-mentioned randomisation method. As per the published intervention protocol, the target sample size for the trial was 140 participants [20]. However, we managed to recruit 121 participants during the study period (Additional file 1). This sample size was large enough to ensure statistical power of 80% in a regression analysis with 15 independent variables (for two-tailed alpha *p* < 0.05 of a regression coefficient), if the true intervention effect in the population was of at least small to medium size (*f* ^2^ < 0.07) according to Cohen [21].

### Study settings

The trial was conducted in purposively selected AHCs located in Dhading, Nuwakot, Kaski, Ramechap, Surkhet, Rolpa and Rupandehi districts. Ayurveda Health Centres (AHCs) are the primary care facilities functioning at the district level in Nepal. Currently, 61 District AHCs are in operation throughout Nepal and they provide basic preventive and curative services. One of the AHC’s regular health promotion programs includes yoga training to school children and senior citizens, to promote health and wellbeing. Between 2015 and 2018, some of the health workers from AHCs were trained in yoga by the Department of Ayurveda and Alternative Medicines. Our study investigators who were also experienced yoga teachers provided training to the same health workers on the intervention package and appointed them as instructors to deliver yoga intervention to the study participants. The instructors were not certified yoga instructors, but they were trained in medical sciences, Ayurveda and yoga for three to six years. The instructors were also permanent public employees, and they agreed to implement the study without altering their usual work routines at the centres. Among the seven trial centres, AHCs located in Kaski, Nuwakot, and Surkhet were able to recruit the initially planned number of participants (*n* = 20) during the study period [20], whereas AHCs from Dhading, Ramechap, Rupandehi, and Rolpa had fewer study participants (Additional file 1).

### Intervention

#### Health Centre based five-day training

The first component of the intervention was a five-day training delivered to the intervention group participants at the trial centres. The participants were invited to attend two-hour yoga training sessions every day for five consecutive days. The instructors (i.e. health care workers from each centre), delivered the yoga training. The wait-list control group participants received the training after the completion of the study.

#### Two hours of health education

In addition to the five-day yoga training session, the participants in the intervention group also received a two-hour health education session. The contents of health education were adopted from the Information, Education and Communication materials endorsed by the National Health Education Information and Communication Center, Ministry of Health and Population, Nepal. The materials contained behavioural and lifestyle modification education targeted to hypertensive participants. The wait-list control group also took part in the health education session.

#### Home-based yoga practice

The intervention group participants were encouraged to practise yoga at home for 30 min per day on five days a week, for the following 90 days from the last day of the training. They were also instructed to visit their trial centres once every 30 days for health assessment and monitoring purposes. The instructors were available over the phone, if the participants needed any help in yoga postures and procedures. The participants were also provided with recorded yoga videos with exercise instructions they could follow if needed.

The yoga program consisted of postures, breathing exercise and meditation structured for 30 min of practice (Additional file 2). Stretching exercise, lateral arc pose and twist pose were included in the initial 9 min of the session. This was followed by breathing exercises for the next 9 min. The remaining 12 min were allocated for meditation and relaxation activities. Evidence suggested that postures (*Asana*) [22–25], breathing exercise (*Pranayam*) [23–26], relaxation [25, 27] and meditation [22, 23, 27] are effective for reducing hypertension while practising them in combination or individually. However, studies found the combination of posture, breathing excise and relaxation/meditation has a greater effect [15, 28]. The yoga session in the current study, therefore, used the combined approach.

The wait-list control group did not receive yoga intervention. They required to visit the trial centres once every 30 days for routine observation.

### Outcomes

SBP at follow up was the primary outcome of the study. Baseline SBP was recorded just before the intervention started and follow-up SBP was measured at 90 ± 5 days counting from the last day of yoga training. We used an aneroid sphygmomanometer (BP AG1–20, Microlife Corp., Taiwan) to record the blood pressure at the Outpatients Department of each trial centre. We initially recorded three blood pressure readings from the participants in each five-minute interval and then averaged the last two readings to get the final measurement. Alongside SBP, we also measured DBP of every individual at baseline and follow-up.

### Data collection

Data were collected by face-to-face interviews, anthropometric measurements and clinical examinations. Blood pressure, body height, body weight and resting heart rate were measured at baseline and follow up. Information on socio-demographic characteristics (age, gender, marital status, ethnicity, education, occupation and income), smoking, alcohol consumption, physical activity and the use of antihypertensive medication were collected before the intervention. We applied structured questionnaires to record socio-demographic characteristics, smoking and alcohol consumption related behaviours, and seven days history of physical activity as previously described in the protocol paper in detail [20]. We measured height and weight using portable stadiometers and digital weighing machines respectively. Radial pulse was taken in the sitting position. Participants were advised to report any change in smoking, alcohol consumption and the use of medications during the study period. The data were collected by the same researcher at baseline and follow-up. The outcome assessors were aware of intervention group allocation.

### Data analysis

The collected data were compiled, edited and entered in Epidata 3.1. We used Stata 16.0 (StataCorp LLC, College Station, TX, USA) to analyse the data. The analysis was performed based on the intention-to-treat (ITT) analysis. To check the distribution of missing data, we created indicator variables for missing outcome variables and dichotomised them on the basis of missing and non-missing. Logistic regression was performed for each indicator variable to check whether missing outcome variables could be predicted by any other study variables. A separate t-test was conducted to check whether the auxiliary variables significantly varied by the missing status of the indicator variables, but none of them was associated with the missingness of data. We used Multiple Imputation by Chained Equations (MICE) model to create 10 imputed datasets (seed of 1234). The imputation included all the variables that were in the estimation model, except income and physical activity. Separate imputation models were built for SBP and DBP. The Fraction of Missing Information (FMI) and Relative Efficiency (RE) were 11.5 and 98.9% for the model with SBP and 6.5 and 99.4% for the model with DBP (Additional files 3 and 4).

Baseline characteristics are presented as absolute frequencies, percentages, medians, arithmetic means and standard deviations. We used mixed-effects linear regression with follow-up SBP and DBP as the outcome variable to analyse the intervention effect. We conducted three analyses (Models 1–3) with a dichotomous independent variable representing belonging to the intervention group (“1”) or control group (“0”). The unstandardized regression coefficient (B) for this dichotomous variable represents the estimated effect of the intervention. In all three models, the trial centre was considered as a second-level variable, and it was allowed to have a random intercept. Other than that, Model 1 (main analysis) was adjusted for baseline outcome measurements (baseline SBP in the analysis with follow-up SBP as the outcome variable and baseline DBP in the analysis with follow-up DBP as the outcome variable). In Model 2, we additionally adjusted for age, gender, marital status, ethnicity, education, occupation, income, smoking, alcohol consumption, physical activity, baseline body mass index (BMI), baseline resting heart rate and antihypertensive medication. For adjusting BMI as a time-varying covariate, along with the variables from Model 2, we added ‘BMI difference’ (calculated as BMI difference = baseline BMI - follow-up BMI) in Model 3.

For sensitivity analysis, we did a complete-case analysis. We simultaneously conducted a mediation analysis to test whether BMI had a significant mediation effect on the outcome. We conducted subgroup analyses based on the level of adherence to the protocol from the trial centre while delivering the intervention. No per-protocol analysis was done, as data on intervention compliance were not available at the individual level. A visual inspection was done to examine whether there was any interaction between covariates and intervention effects, and their average marginal (partial) effects were plotted in the marginal plots. All tests were two-tailed and *p* < 0.05 was considered to indicate statistical significance.

### Fidelity assessment of the trial

We conducted a post-intervention survey to retrospectively assess the fidelity of intervention. Fidelity assessment was performed in three domains: intervention delivery (i.e. whether the contents of the intervention were delivered in line with the protocol), intervention receipt (i.e. whether participants understood or learned intervention components) and intervention enactment (i.e. whether participants were able to perform home-based yoga practice as instructed) [29, 30]. For this purpose, information on the contents delivered during the five-day yoga training session was collected by a thorough review of the training documents, attendance sheets and log-books from each centre. The collected information was then used to calculate the actual score of content delivery using a checklist (Additional file 5). The checklist contains the names of 10 structured yoga items and 5 health education topics that were to be covered during the intervention. Covering each yoga item and health education topic was assigned one point, so the maximum total score was 15 points. If the trial centre included any additional yoga items other than the ones specified in the protocol they were given a negative point. The level of agreement between the contents that were actually delivered and those that were supposed to be delivered was assessed using the percentage of agreement (PoA), expressed as the ratio of the actual score of the items and topics covered in the training session (numerator) and the total score of all yoga items and health education topics as per the protocol (denominator). The percentage was expected to be at least 90% for each centre [31].

Similarly, to assess whether the participants learned the proper yoga skills during the training sessions (i.e. intervention receipt) and applied the same skills while practising at home (i.e. intervention enactment), we interviewed randomly selected 20% of participants from the intervention group using a structured questionnaire. The participants were asked whether the training provided by the instructor was sufficient for them to learn yoga properly and whether they had practised yoga at home as instructed by the trainer (i.e. as per protocol). The responses were provided on a scale from 1 to 5, with 1 indicating low and 5 indicating high sufficiency of the training. The findings of the survey guided the sensitivity analysis.

### Data and safety monitoring

A clinical doctor who led the data monitoring and quality assurance team monitored the implementation of the trial, including participant recruitment and intervention delivery. The doctor provided necessary feedback to the study team. He was also responsible for reviewing data safety and quality, and he was the first person to report missing information and errors during data collection.

Regarding participants’ safety, participants were instructed to report any serious adverse events during the intervention to the researchers located in each district. These researchers together with the clinicians from their centres were responsible for reviewing and responding to any reported adverse event and for reporting it to the Principal Investigator and Ethical Review Board.

## Results

### Intervention effects

**Fig. 1:**
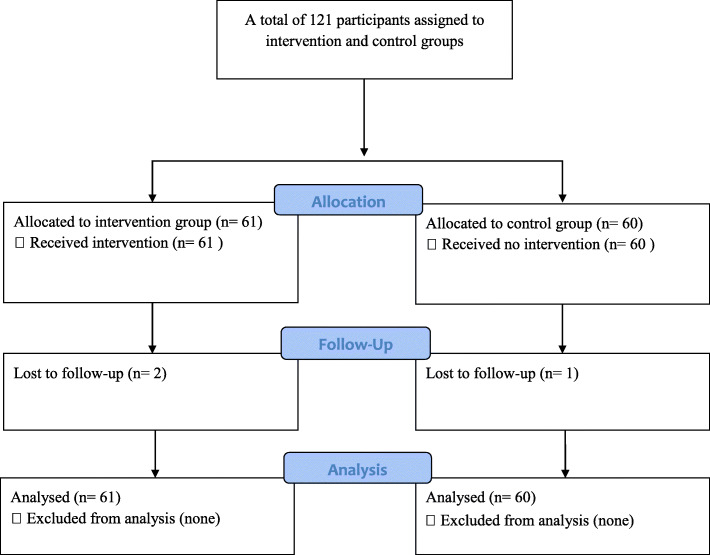
Participant flow diagram

Data on the primary outcomes were available for a total of 118 participants (Fig. 1). Three participants, two from intervention group (males) and one (female) from control group were lost to follow-up. None of the participants reported any changes in medication, tobacco use and alcohol consumption during the study period. Participants also did not report experiencing any adverse events as a result of the intervention.

Fifty-two percent of the participants were males. The mean (standard deviation [SD]) age of the participants was 47.7 (10.7) years. The median (interquartile range [IQR]) of years of formal education was 5 (11), where more than one third (35.5%) of participants had no formal schooling. Smoking and alcohol consumption were reported by 14.9 and 29.7% of participants, respectively (Table 1).

**Table 1:** Baseline characteristics of the participants

Characteristics		Intervention group	Control group
		*n* (%)^a^ or mean (SD) ^b^ or median (IQR) ^c^	*n* (%)^a^ or mean (SD) ^b^ or median (IQR)^c^
Age (years)		47.1 (11.0)	48.4 (10.7)
Gender	Female	35 (57.4)	23 (38.3)
	Male	26 (42.6)	37 (61.6)
Marital status	Married	55 (90.2)	56 (93.3)
	Others (unmarried, widow)	6 (9.8)	4 (6.7)
Ethnicity	Brahman	17 (27.9)	21 (35.0)
	Chhetri	13 (21.3)	12 (20.0)
	Janajati	25 (41.0)	21 (35.0)
	Others	6 (9.8)	6 (10.0)
Education (years)		5 (11)	6 (11.5)
Occupation	Paid job	15 (24.6)	9 (15.0)
	Self-employed	20 (32.8)	25 (41.7)
	Homemaker	24 (39.3)	16 (26.7)
	Others	2 (3.3)	10 (16.6)
Annual household income (Nepalese rupees)		300,000 (400000)	200,000 (325000)
Smoking	Yes	7 (11.5)	11 (18.3)
	No	54 (88.5)	49 (81.7)
Alcohol consumption	Yes	13 (21.3)	23 (38.3)
	No	48 (78.7)	37 (61.7)
Physical activity (MET-minutes/week)		1800 (2340)	1530 (2580)
Body mass index (kg/m^2^)		27.3 (4.7)	27.5 (5.0)
Resting heart rate (beats per minute)		77.4 (5.1)	77.6 (6.1)
Systolic blood pressure (mmHg)		141. 7 (9.1)	136.9 (9.0)
Diastolic blood pressure (mmHg)		90.3 (5.4)	89.4 (5.1)
Antihypertensive medication	Yes	35 (57.4)	36 (60.0)
	No	26 (42.6)	24 (40.0)

^a^ mean and standard deviation (SD) were shown for age, body mass index, resting heart rate, systolic blood pressure and diastolic blood pressure

^b^ number (*n*) and percentage (%) for gender, marital status, ethnicity, occupation, smoking, alcohol consumption and antihypertensive medication

^c^ median and interquartile range (IQR) are given for education, annual household income and physical activity (moderate and vigorous activities)

The median (IQR) physical activity level expressed in MET-minutes per week was 1800 (2340) and 1530 (2580) for the intervention and control group respectively. The mean (SD) BMI was 27.4 (4.8) in kg/m^2^. The mean (SD) of baseline SBP was 141.7 (9.1) mmHg in the intervention group and 136.9 (9.0) mmHg in the control group (Table 1). More than half (58.7%) of the participants were on antihypertensive medications.

**Fig. 2:**
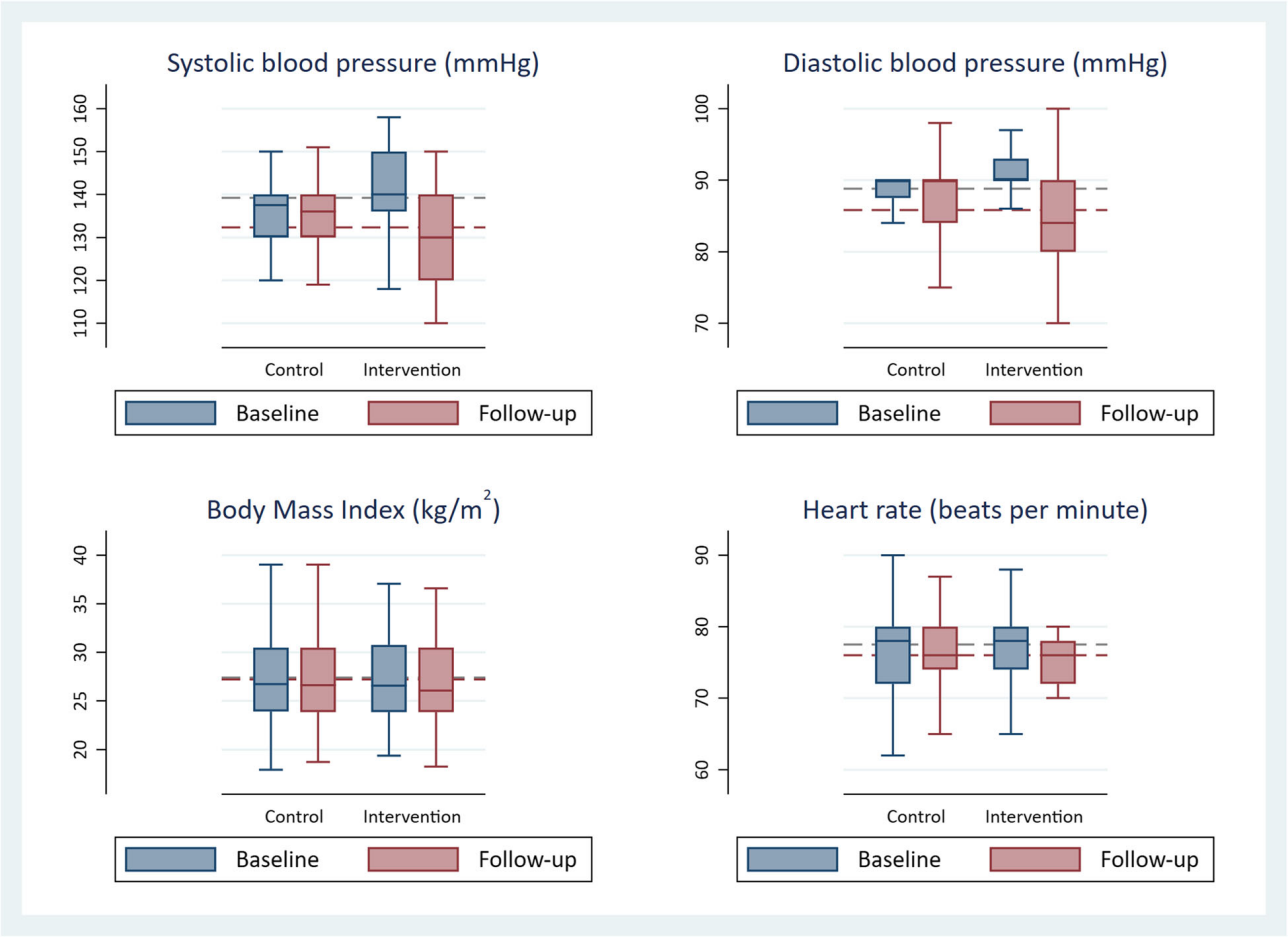
Baseline and follow-up blood pressure, BMI and resting heart rate. Note: Grey dash line = Baseline mean; Maroon dash line = Follow-up mean; Box represents the inter-quartile range

At follow-up, the mean (SD) of SBP was 130.1 (9.3) mmHg and 134.6 (11.0) mmHg in intervention and control group, respectively. The mean of post-intervention DBP was 84.1(6.3) mmHg in the intervention group and 87.6 (7.0) mmHg in the control group (Fig. 2).

The average reduction in SBP in the intervention and control group was 11.5 mmHg and 2.2 mmHg respectively (Table 2). The mean reduction in DBP in the intervention and control group was 6.1 mmHg and 1.9 mmHg. Compared to baseline BMI and resting heart rate, the follow-up BMI and resting heart rate in the intervention group decreased by 0.37 kg/m^2^ (vs 0.7 kg/m^2^ in control group) and 1.9 beats per minute (vs 1.2 beats per minute in control group), respectively.

**Table 2:** Changes in outcome variables from baseline to follow-up

Characteristics		Change in SBP^a^		Change in DBP^b^	
		Intervention group	Control group	Intervention group	Control group
		mean	mean	mean	mean
Total		11.5	2.3	6.1	1.8
Gender	Female	10.1	5.0	5.3	3.9
	Male	13.5	0.6	7.2	0.6
Marital status	Married	11.5	1.8	6.0	1.7
	Others (unmarried, widow)	10.8	8.3	7.0	3.8
Ethnicity	Brahman	13.8	−0.5	7.6	0.0
	Chhetri	8.5	7.4	3.3	5.0
	Janajati	11.3	3.2	6.5	2.4
	Others	12.3	−0.8	6.7	0.3
Occupation	Paid job	11.0	−0.4	5.1	−0.6
	Self-employed	13.5	1.0	7.9	2.4
	Homemakers	9.7	7.1	5.3	4.7
	Others	16.5	0.5	5.0	−1.6
Smoking	No	11.3	1.9	5.9	2.0
	Yes	12.7	3.9	7.5	1.1
Alcohol consumption	No	11.3	3.4	5.9	2.8
	Yes	12.2	0.4	7.0	0.3
Antihypertensive Medication	No	14.0	2.0	7.3	1.0

^a^ systolic blood pressure

^b^ diastolic blood pressure

In the main analysis, being in the intervention group was associated with an average 7.66 mmHg (95% CI: 4.93, 10.4) greater reduction in SBP between baseline and follow-up, compared to the control group (Table 3: Model 1). After adjusting for age, gender, ethnicity, marital status, education, occupation, household income, smoking, alcohol consumption, physical activity, baseline body mass index, antihypertensive medication, baseline resting heart rate and baseline SBP (Table 3: Model 2), being in the intervention group was associated with an average 7.41 mmHg (95% CI: 5.06, 9.76) greater reduction in SBP between baseline and follow-up, compared to the control group. In Model 3, the change in BMI from baseline to follow-up was significantly associated with SBP (B = − 2.49 mmHg, 95% CI: − 3.74, − 1.20; Additional file 6). The unstandardized regression coefficient (B) representing the effect of the intervention in Model 3 was − 6.36 (95% CI: − 8.63, − 4.10), again favouring the intervention group (Table 3). Detailed outputs of Model 2 and Model 3 are provided in Additional files 6 and 7.

**Table 3:** Intervention effects: results of multilevel mixed-effects linear regression

Outcome variable	Model 1^a^		Model 2^b^		Model 3^c^	
	B^d^	95% CI^e^	B^d^	95% CI^e^	B^d^	95% CI^e^
Systolic blood pressure	−7.66^***^	−10.4, −4.93	− 7.41^***^	−9.76, −5.06	−6.36^***^	−8.63, − 4.10
Diastolic blood pressure	−3.86^**^	− 6.65, − 1.06	−3.49^**^	−6.13, − 0.86	−2.73^*^	−5.06, − 0.41

^a^ Model included a dichotomous independent variable representing belonging to the intervention group (“1”) or control group (“0”) and trial centre as a second-level variable, and was adjusted for baseline systolic or diastolic blood pressure (depending on the outcome variable)

^b^ Adjusted for age, gender, marital status, ethnicity, education, occupation, income, smoking, alcohol consumption, physical activity, body mass index (BMI), resting heart rate, and baseline systolic or diastolic blood pressure (depending on the outcome variable)

^c^ Additionally adjusted for the difference in BMI between baseline and follow-up

^d^ Unstandardized regression coefficient

^e^ 95% confidence interval for B

^*^ *p* < 0.05

^**^ *p* < 0.01

^***^ *p* < 0.001

The reduction in DBP between baseline and follow-up was 3.86 (95% CI: 1.06, 6.65) units higher for the intervention group, compared to the control group. In Model 2, we also found that being in the intervention group was associated with on average 3.49 mmHg (95% CI: 0.86, 6.13) greater reduction in DBP between baseline and follow-up, compared with being in the control group. The unstandardized regression coefficient (B) representing the effect of the intervention in Model 3 was − 2.73 (95% CI: − 5.06, − 0.41), again favouring the intervention group (Additional file 7).

The regression coefficients in the complete-case, sensitivity analysis (using Model 1) were nearly the same as in the main analysis (− 7.62 vs. -7.66 for SBP and − 3.88 vs. -3.86 for DBP.). In mixed-effects mediation analyses, the mediating effects of the change in BMI from baseline to follow-up on the primary intervention outcomes were not significant.

The change in resting heart rate from baseline to follow-up was not significantly associated with SBD and DBP (Additional file 8). The marginal plots for gender, medication status, smoking and alcohol consumption by intervention allocation did not visually show any sign of interaction effect (Additional file 9).

### Findings from fidelity assessment

The PoA was 100% for five health centres and 90% for the two remaining centres. The difference in PoA between the health centres was not significantly associated with the primary intervention outcomes. The average score for intervention receipt was 4.3 out of five (the score provided by the participants in response to the question on whether the training was sufficient for them to learn yoga or not; Additional file 10). Similarly, 100% of the participants reported they could perform home-based yoga practice in the same way as they were trained to do by the yoga instructor.

## Discussion

In this multicentre randomised controlled trial, we found that a 3-month yoga intervention reduces systolic and diastolic blood pressure among hypertensive patients. This implies that yoga programmes can be promoted through primary care settings as an effective non-pharmacological therapy to treat hypertension.

Our findings are consistent with a recent systematic review that found an average reduction of SBP by 7.9 mmHg and DBP by 4.3 mmHg among the participants who received a yoga intervention including breathing techniques and meditation [15]. In another review, Cramer et al. found that yoga interventions lasting eight weeks or more, reduced SBP on average by − 9.65 mmHg [32]. The pooled effect from the Cramer et al. meta-analysis may seem somewhat higher than the average effect found in our study. However, due to a relatively small pooled sample size and large heterogeneity between individual studies included in the meta-analysis, the confidence interval of the pooled effect from Cramer et al. [32] study was very wide, and it largely overlaps with our narrower confidence interval for the respective effect in our study. A smaller blood pressure-lowering effect in our study compared to the Cramer et al. [32] meta-analysis might be because of the attenuation of the intervention effect due to its implementation in a real-world clinical setting. Likewise, the implementation of yoga intervention in our study was done by health workers. It might be that the effect of yoga on blood pressure reduction would be higher, if the intervention was implemented by certified yoga instructors or kinesiologists.

Studies have investigated several possible underlying mechanisms for clinical effects of yoga on hypertension [33–35]. One of the hypothesized mechanisms is that yoga affects the autonomic nervous system by stimulating activity of parasympathetic and reducing activity of sympathetic nervous system [33]. It is also postulated that yoga increases bioavailability and blood levels of nitric oxide and promotes vasodilation [33]. Additionally, participation in yoga as a “mind-body” activity has been associated with improved physiological markers, reduced symptoms of stress, and better mood [36, 37]. Pascoe et al. [36] in their systematic review concluded that mindfulness-based activities, including yoga, lead to decreased cortisol level, a stress hormone that has been linked to high blood pressure. Thoroughly investigating the mechanism of the effect of yoga on blood pressure was beyond the scope of this study. Nevertheless, we considered the possible mediating effect of the change in BMI and resting heart rate between baseline and follow-up, and we found no strong indication either of these would constitute the underlying mechanism. Given that yoga is a complex activity, it might be challenging to determine a single mechanism that would explain antihypertensive effects of all components of yoga. Therefore, to illuminate the underlying causal pathways, future studies will need to assess in detail different physiological, biomedical and stress biomarkers in relation to specific yoga components.

The main strength of the current study was that the intervention was evaluated in a real-world clinical setting. The number of such studies is generally limited. Moreover, to the best of our knowledge, this was the first study that investigated the effects of a primary health care staff-led yoga intervention on high blood pressure among the patients attending public health centres in a low-income country. One of the benefits of conducting the trial in a real-world setting is that the study could have good external validity and it could enhance the likelihood that it is translated into practice [18, 19]. The current study has the potential to be scaled up nationwide in Nepal, as the remaining AHCs are also equipped with both physical and human resources to implement yoga intervention. The situation is likely to be similar in many other LMICs. In Nepal, the national policy and mechanisms of using yoga as a health promotion tool are also already in place. The Multisectoral Action Plan for Prevention and Control of NCDs (2014–2020) and Urban Health Policy (2015) integrated yoga as a strategy for NCD prevention and control. Similarly, the Department of Ayurveda and Alternative Medicines have launched yoga-based interventions such as ‘*Swatha Jiwan karyakram’*(informal translation: Healthy Life Program) and ‘*Vidhaylaya yoga shiskya karyakram’* (informal translation: School Yoga Education Program) in 75 districts of Nepal to promote health and wellbeing of elderly and school children. The current intervention could also be an economically viable approach, as it can utilize existing resources and can also be integrated into the ongoing program that has similar modalities, such as ‘*Swatha Jiwan karyakram’*. However, further studies are required to test the cost-effectiveness of upscaling the program. Furthermore, the current study also had well-structured intervention packages comprising different components of yoga, including postures, breathing exercises, relaxation and meditation. Previous evidence showed that these components in combination were likely to have a better positive impact on health than individual components [15, 28]. Likewise, the session timing (i.e. 30 min) and frequency (i.e. five sessions per week) were selected to be in line with the World Health Organization physical activity guidelines (i.e. 150 or more minutes a week of moderate-to-vigorous physical activity). This study had a shorter session timing compared to previous studies in which the average session time was 59.2 min [15]. This might have positively affected participant compliance. Lastly, as this trial was conducted in several centres, representing large geographical areas of Nepal, the findings could be generalized beyond the trial participants and centres.

The current study has some limitations. Firstly, hypertension was diagnosed based on blood pressure measured on two occasions only that were 1–2 weeks apart. Although most participants were previously diagnosed hypertensive patients, it might be that we misclassified some of the newly diagnosed participants. We did not manage to collect information on r adherence to the study protocol from all participants. Evidence shows that the effect of yoga may vary depending on the frequency and duration of yoga practice [15]. Future studies on the effects of yoga on blood pressure should aim to collect such data, to enable conducting per-protocol analysis. Furthermore, the post-intervention blood pressure measurements were not done on the same day for all participants, as this was not feasible. It was measured between the 85th and the 95th day of the intervention, as not all participants were available for the follow-up measurement on the 90th day. Besides, the variation in the level of yoga competency of the health workers who provided training to the participants might have also influenced the study outcomes. Likewise, the pre and post-intervention data were collected by the same persons and they used aneroid blood pressure machines to assess blood pressure. That might have introduced rater bias. We did not assess long-term effects of the intervention. It might be that the intervention would not be as efficient and sustainable over a longer period, as participant compliance to the protocol would likely reduce over time. Lastly, as the study included only first-stage hypertensive patients, study findings cannot be generalised to patients with higher stages of hypertension.

## Conclusion

A simple, 3-month yoga intervention delivered by health workers in primary care centres and coupled with home-based practice is effective in lowering high blood pressure among hypertensive patients. Given that the study was conducted in real-world clinical settings, our findings suggest the intervention strategy should be considered as adjuvant or initial lifestyle therapy for hypertension in primary care.

## Supplementary Information

**Additional file 1.** Number of participants across trial centres.

**Additional file 2.** Yoga module.

**Additional file 3.** Imputation variance information for systolic blood pressure.

**Additional file 4.** Imputation variance information for diastolic blood pressure.

**Additional file 5.** Checklist for fidelity assessment.

**Additional file 6.** Intervention effects on systolic blood pressure.

**Additional file 7.** Intervention effects on diastolic blood pressure.

**Additional file 8.** Models adjusted for the difference in heart rate between baseline and follow-up.

**Additional file 9.** Marginal plots.

**Additional file 10.** Findings from fidelity assessment.

## Data Availability

The datasets and study materials will be available from the corresponding author on request.

## Abbreviations

AHC: Ayurveda Health Centre
BMI: Body mass index
DBP: Diastolic blood pressure
ERB: Ethical Review Board
ITT: Intention to treat
IQR: Interquartile range
MET: Metabolic Equivalent
NCD: Non-communicable disease
SBP: Systolic blood pressure
SD: Standard deviation
