# Supplementary material for: Effects of a health worker-led 3-month yoga intervention on blood pressure of hypertensive patients: a randomised controlled multicentre trial in the primary care setting
Source: BMC Public Health. 2021 Mar 20;21:550. doi: 10.1186/s12889-021-10528-y (PMC7981931; doi:10.1186/s12889-021-10528-y)
Supplement: Supplementary file 1 — Additional file 1. Number of participants across trial centres. [file 12889_2021_10528_MOESM1_ESM.pdf]

Additional file 1. Number of participants across trial centres

	Intervention group		Control group	
**Trial centre**	**Target sample size**	**Recruited**	**Target sample size**	**Recruited**
Dhading	10	9	10	8
Kaski	10	10	10	10
Nuwakot	10	10	10	10
Ramechap	10	9	10	10
Surkhet	10	10	10	10
Rolpa	10	5	10	4
Rupandehi	10	8	10	8
Total	**70**	**61**	**70**	**60**
