# Supplementary material for: Effects of a health worker-led 3-month yoga intervention on blood pressure of hypertensive patients: a randomised controlled multicentre trial in the primary care setting
Source: BMC Public Health. 2021 Mar 20;21:550. doi: 10.1186/s12889-021-10528-y (PMC7981931; doi:10.1186/s12889-021-10528-y)
Supplement: Supplementary file 2 — Additional file 2. Yoga module. [file 12889_2021_10528_MOESM2_ESM.pdf]

Additional file 2. Yoga Module for Hypertensive Patients

	**Practices**	**Duration** (min)
1.	Starting with "omkar"	1 min
2.	Warm-up exercises with synchronization of breathing in the sitting position:<br>1. Toe bending<br>2. Ankle bending<br>3. Knee bending<br>4. Half butterfly<br>5. Finger bending<br>6. Wrist bending<br>7. Elbow bending<br>8. Shoulder rotation<br>9. Neck bending up and down	5 min
3.	Yogic abdominal awareness, breathing and feeling in "shavasana"	3 min
4.	"Ardhakatichakrasan" (Lateral Arc Pose)	2 min
5.	"Vakrasana" (Twist Pose)	2 min
6.	"Chandravedi Pranayam" (Left nostril breathing)	2 min
7.	"Sheetali Pranayam" (Cooling breathing)	2 min
8.	"Nadi Suddhi Pranayama" (Alternate Nostril breathing)	2 min
9.	"Bhramari" (Humming bee breathing)	2 min
10.	Yoga "nidra" (Yogic sleep)	9 min
	**Total Time**	**30 min**
