# Supplementary material for: Effects of a health worker-led 3-month yoga intervention on blood pressure of hypertensive patients: a randomised controlled multicentre trial in the primary care setting
Source: BMC Public Health. 2021 Mar 20;21:550. doi: 10.1186/s12889-021-10528-y (PMC7981931; doi:10.1186/s12889-021-10528-y)
Supplement: Supplementary file 3 — Additional file 3. Imputation variance information for systolic blood pressure. [file 12889_2021_10528_MOESM3_ESM.pdf]

Additional file 3. Imputation variance information for systolic blood pressure

	Imputation variance					
**Variables**	**Within**	**Between**	**Total**	**RVI**	**FMI**	**Relative efficacy**
Age	0.009826	0.000038	0.009868	0.004202	0.004188	0.999581
Female	2.8767	0.11946	3.0081	0.045679	0.044089	0.99561
Ethnicity						
Chhetri	4.41951	0.353281	4.80812	0.08793	0.082155	0.991851
Janajati	4.87066	0.12106	5.00382	0.02734	0.026766	0.997331
Others	0.852885	0.171985	1.04207	0.221815	0.187475	0.981597
Marital status						
Others	4.36113	0.420532	4.82371	0.10607	0.09774	0.990321
Education	0.014923	0.000657	0.015646	0.048433	0.046648	0.995357
Occupation						
Self employed	1.96841	0.253266	2.247	0.141532	0.126961	0.987463
Homemakers	5.54746	0.112635	5.67136	0.022334	0.02195	0.99781
Others	4.95901	0.135234	5.10776	0.029997	0.029307	0.997078
Income	6.70E-14	5.30E-16	6.70E-14	0.008675	0.008617	0.999139
Smoking						
Yes	4.07474	0.232305	4.33028	0.062712	0.059739	0.994062
Alcohol consumption						
Yes	1.93501	0.172866	2.12516	0.09827	0.091092	0.990973
Physical activity	2.50E-08	1.60E-09	2.60E-08	0.072499	0.068544	0.993192
BMI baseline	0.025952	0.001298	0.02738	0.055023	0.052726	0.994755
BMI difference	0.378505	0.036402	0.418548	0.105791	0.097504	0.990344
Antihypertensive medication						
yes	1.92209	0.063736	1.9922	0.036476	0.035457	0.996467
Heart rate	0.022505	0.000172	0.022695	0.008409	0.008355	0.999165
SBP baseline	0.009674	0.000079	0.009762	0.009015	0.008952	0.999106
Group						
Intervention	**1.17849**	**0.137016**	**1.3292**	**0.127891**	**0.115912**	**0.988542**
_cons	161.666	0.396209	162.101	0.002696	0.00269	0.999731
