# Supplementary material for: Effects of a health worker-led 3-month yoga intervention on blood pressure of hypertensive patients: a randomised controlled multicentre trial in the primary care setting
Source: BMC Public Health. 2021 Mar 20;21:550. doi: 10.1186/s12889-021-10528-y (PMC7981931; doi:10.1186/s12889-021-10528-y)
Supplement: Supplementary file 4 — Additional file 4. Imputation variance information for diastolic blood pressure. [file 12889_2021_10528_MOESM4_ESM.pdf]

Additional file 4. Imputation variance information for diastolic blood pressure

	Imputation variance					
**Variables**	**Within**	**Between**	**Total**	**RVI**	**FMI**	**Relative efficacy**
Age	0.004395	8.60E-06	0.004405	0.002142	0.002138	0.999786
Female	0.770965	0.035424	0.809931	0.050543	0.0486	0.995163
Ethnicity						
Chhetri	0.428518	0.246589	0.699766	0.632992	0.407099	0.960883
Janajati	1.82056	0.066194	1.89338	0.039995	0.038773	0.996138
Others	2.63757	0.054279	2.69727	0.022637	0.022243	0.997781
Marital status						
Others	0.538028	0.242699	0.804997	0.496198	0.347397	0.966427
Education	0.004524	0.000358	0.004918	0.087043	0.081381	0.991928
Occupation						
Self employed	0.528303	0.075803	0.611685	0.157832	0.139861	0.986207
Homemakers	0.887174	0.044395	0.936008	0.055045	0.052746	0.994753
Others	1.80484	0.033393	1.84157	0.020352	0.020033	0.998001
Income	4.70E-14	9.70E-16	4.80E-14	0.022573	0.022181	0.997787
Smoking						
Yes	1.22855	0.213053	1.46291	0.19076	0.164949	0.983773
Alcohol consumption						
Yes	0.816512	0.098516	0.924879	0.13272	0.11985	0.988157
Physical activity	7.90E-08	5.20E-10	8.00E-08	0.007204	0.007164	0.999284
BMI baseline	0.018207	0.000166	0.018389	0.010022	0.009944	0.999007
BMI difference	0.33941	0.013492	0.354251	0.043726	0.042267	0.995791
Antihypertensive medication						
yes	1.11671	0.019882	1.13858	0.019585	0.019289	0.998075
Heart rate	0.00486	0.000106	0.004977	0.023952	0.02351	0.997655
SBP baseline	0.016487	0.000852	0.017425	0.056865	0.054413	0.994588
Group						
Intervention	1.30839	0.086797	1.40387	0.072973	0.068967	0.993151
_cons	74.1395	4.59098	79.1895	0.068116	0.064617	0.99358
