# Supplementary material for: Effects of a health worker-led 3-month yoga intervention on blood pressure of hypertensive patients: a randomised controlled multicentre trial in the primary care setting
Source: BMC Public Health. 2021 Mar 20;21:550. doi: 10.1186/s12889-021-10528-y (PMC7981931; doi:10.1186/s12889-021-10528-y)
Supplement: Supplementary file 5 — Additional file 5. Checklist for fidelity assessment. [file 12889_2021_10528_MOESM5_ESM.pdf]

Additional file 5. Checklist for the assessment of content fidelity

	**Yoga exercises**	**Score**	**Health education topics**	**Score**
1.	"Omkar"	1	Assessing the level of participants' understanding of high blood pressure	1
2.	Warm-up exercises	1	Introduction about high blood pressure	1
3.	Yogic abdominal awareness	1	Risk factors for high blood pressure	1
4.	Lateral arc pose	1	Complications of high blood pressure	1
5.	Twist pose	1	Behavioural approaches for the management of high blood pressure	1
6.	Left nostril breathing	1		
7.	Cooling breathing	1		
8.	Alternate nostril breathing	1		
9.	Humming bee breathing	1		
10.	Yogic sleep	1		
	**Maximum score**	**10**		**5**
