# Supplementary material for: Effects of a health worker-led 3-month yoga intervention on blood pressure of hypertensive patients: a randomised controlled multicentre trial in the primary care setting
Source: BMC Public Health. 2021 Mar 20;21:550. doi: 10.1186/s12889-021-10528-y (PMC7981931; doi:10.1186/s12889-021-10528-y)
Supplement: Supplementary file 6 — Additional file 6. Intervention effects on systolic blood pressure. [file 12889_2021_10528_MOESM6_ESM.pdf]

Additional file 6. Intervention effects on systolic blood pressure: results of multilevel mixed-effects linear regression

Characteristic		Unadjusted model†		Model 2‡		Model 3§	
		B	CI‖	B	CI‖	B	CI‖
Age (years)		0.1	-0.10, 0.31	0.06	-0.15, 0.28	0.14	-0.06, 0.33
Gender	Male						
	Female	0.06	-5.21, 5.34	1.81	-1.70, 5.32	2.86	-0.54, 6.26
Ethnicity	Brahman						
	Chhetri	-0.58	-5.78, 4.62	-1.51	-5.90, 2.88	-0.35	-4.65, 3.95
	Janajati	1.04	-3.47, 5.56	-0.88	-5.15, 3.39	-0.99	-5.37, 3.39
	Others	5.16	-0.04, 10.37	2.78*	0.12, 5.44	3.72***	1.71, 5.73
Marital status	Married						
	Others	1.19	-1.86, 4.23	-0.72	-5.83, 4.39	-1.02	-5.33, 3.29
Education		-0.05	-0.42, 0.31	-0.01	-0.24, 0.23	0.05	-0.19, 0.30
Occupation	Job						
	Self-employed	-1.71	-5.88, 2.47	-2.85	-5.79, 0.09	-2.01	-4.96, 0.93
	Homemaker	-2.03	-6.66, 2.59	-3.23	-7.95, 1.49	-3.41	-8.08, 1.25
	Others	2.7	-2.53, 7.92	-0.95	-5.49, 3.58	-0.38	-4.81, 4.05
Household income (Nepali Rupees)		-1.10e-06	-1.90e-06 -2.89e-07	-7.58e-07 ***	-1.09e-06, -4.28e-07	-9.93e-07***	-1.50e-06, -4.85e-07
Smoking	No						
	Yes	3.2	-2.65, 9.06	0.08	-4.62, 4.78	-0.98	-5.06, 3.10
Alcohol consumption	No						
	Yes	1.55	-1.86, 4.96	1.1	-1.51, 3.70	1.52	-1.34, 4.38
Physical activity (METs-minute)		0.00034	-0.0006, 0.0013	-0.00028	-0.0007, 0.00014	-0.00007	-0.00039 0.00025
Baseline BMI (kg/m^2)		0.06	-0.23, 0.34	0.35*	0.04, 0.66	0.36*	0.04, 0.69
Difference in BMI (kg/m^2)						-2.47***	-3.74, -1.20
Antihypertensive medication	No						
	Yes	-0.46	-3.96, 3.04	-0.08	-2.87, 2.70	-0.93	-3.70, 1.83
Baseline heart rate (beats/minute)		-0.01	-0.37, 0.35	-0.05	-0.34, 0.25	-0.02	-0.31, 0.28
Baseline systolic blood pressure (mmHg)		0.54***	0.36, 0.72	0.67***	0.45, 0.90	0.63***	0.43, 0.82
**Treatment allocation**	Control						
	Intervention	-4.53	-9.88, -0.73	-7.41***	-9.76, -5.06	-6.36***	-8.63, -4.10

Note: †, Model included a dichotomous independent variable representing belonging to the intervention group ("1") or control group ("0") and trial centre as a second-level variable;
‡, Adjusted for age, gender, marital status, ethnicity, education, occupation, income, smoking, alcohol consumption, physical activity, body mass index (BMI), resting heart rate, and baseline systolic blood pressure;
§, Additionally adjusted for the difference in BMI between baseline and follow-up;
‖, 95% confidence interval for B;
*, p <0.05; **, p <0.01; ***, p<0.001
