# Supplementary material for: Effects of a health worker-led 3-month yoga intervention on blood pressure of hypertensive patients: a randomised controlled multicentre trial in the primary care setting
Source: BMC Public Health. 2021 Mar 20;21:550. doi: 10.1186/s12889-021-10528-y (PMC7981931; doi:10.1186/s12889-021-10528-y)
Supplement: Supplementary file 7 — Additional file 7. Intervention effects on diastolic blood pressure. [file 12889_2021_10528_MOESM7_ESM.pdf]

Additional file 7.. Intervention effects on diastolic blood pressure: results of multilevel mixed-effects linear regression

**Characteristic**		**Unadjusted model** †		**Model 2**‡		**Model 3**§	
		**B**	**CI**‖	**B**	**CI**‖	**B**	**CI**‖
Age (years)		0.04	-0.07,0.15	0.04	-0.10, 0.17	0.11	-0.02, 0.24
Gender	Male						
	Female	-0.71	-3.62, 2.20	-0.13	-2.01, 1.74	0.71	-1.06, 2.47
Ethnicity	Brahman						
	Chhetri	1.62	-1.51, 4.74	0.7	-1.48, 2.88	1.44	-0.23, 3.12
	Janajati	0.22	-3.09, 3.54	-0.37	-2.96, 2.21	-0.68	-3.38, 2.01
	Others	1.96	-2.31, 6.24	1.99	-1.54, 5.52	2.57	-0.65, 5.79
Marital status	Married						
	Others	1.14	-1.03, 3.32	-1.41	-4.31, 1.50	-1.58	-3.36, 0.21
Education		0.06	-0.14,0.26	-0.06	-0.23, 0.12	-0.01	-0.15, 0.13
Occupation	Job						
	Self-employed	-3.71*	-7.13, -0.3	-3.83***	-5.71, -1.95	-3.03***	-4.56, -1.49
	Homemaker	-2.44	-5.73, 0.84	-2.32*	-4.38, -0.26	-2.39*	-4.28, -0.49
	Others	1.01	-3.11, 5.13	0.67	-2.15, 3.48	1.07	-1.59, 3.73
Household income (Nepali Rupees)		-6.33e-07	-1.09e-06, -1.74e-07	-5.53e-07***	-8.56e-07, -2.49e-07	-7.61e-07 ***	-1.19e-06, -3.30e-07
Smoking	No						
	Yes	1.62	-1.86, 5.09	0.02	-4.02, 4.05	-1.15	-3.53, 1.23
Alcohol consumption	No						
	Yes	1.42	-1.17, 4.0	0.83	-1.07, 2.72	1.19	-0.70, 3.08
Physical activity (METs-minute)		0.00031	-0.0004, 0.00104	-1.51e-06	-.000782, 0.00078	0.00017	-0.00038, 0.00073
Baseline BMI (kg/m^2)		-0.04	-0.22,0.14	0.04	-0.19, 0.27	0.08	-0.18, 0.35
Difference in BMI (kg/m^2)						-2.33***	-3.49, -1.16
antihypertensive medication	No						
	Yes	-0.55	-2.78, 1.68	-0.2	-2.53, 2.12	-1.14	-3.23, 0.95
Baseline heart rate (beats/minute)		0.1	-0.12,0.32	0.08	-0.07, 0.23	0.1	-0.04, 0.24
Baseline diastolic blood pressure (mmHg)		0.41**	0.15,0.67	0.42**	0.13, 0.70	0.41**	0.15, 0.67
Treatment allocation	Control						
	Intervention	-3.49*	-6.4, -0.59	-3.49**	-6.13, -0.86	-2.73*	-5.06, -0.41

Note: †, Model included a dichotomous independent variable representing belonging to the intervention group ("1") or control group ("0") and trial centre as a second-level variable;
‡, Adjusted for age, gender, marital status, ethnicity, education, occupation, income, smoking, alcohol consumption, physical activity, body mass index (BMI), resting heart rate, and baseline diastolic blood pressure;
§, Additionally adjusted for the difference in BMI between baseline and follow-up;
‖, 95% confidence interval for B;
*, p <0.05; **, p <0.01; ***, p<0.001
