# Supplementary material for: Effects of a health worker-led 3-month yoga intervention on blood pressure of hypertensive patients: a randomised controlled multicentre trial in the primary care setting
Source: BMC Public Health. 2021 Mar 20;21:550. doi: 10.1186/s12889-021-10528-y (PMC7981931; doi:10.1186/s12889-021-10528-y)
Supplement: Supplementary file 8 — Additional file 8. Models adjusted for the difference in heart rate between baseline and follow-up. [file 12889_2021_10528_MOESM8_ESM.pdf]

Additional file 8. Models adjusted for the difference in heart rate between baseline and follow-up

Table 1. Intervention effects on systolic blood pressure

Characteristic		Model 4†	
		**B**	**CI§**
Age (in years)		0.15	-0.06, 0.36
Gender	Male		
	Female	2.94	-0.59, 6.47
Ethnicity	Brahman		
	Chhetri	-0.57	-4.87, 3.72
	Janajati	-0.95	-5.38, 3.48
	Others	3.95**	1.18, 6.72
Marital status	Married		
	Others	-0.82	-4.87, 3.22
Education		0.07	-0.17, 0.32
Occupation	Job		
	self-employed	-2.01	-4.69, 0.68
	Homemaker	-3.41	-7.95, 1.13
	Others	-0.55	-5.05, 3.95
Household income (Nepali Rupees)		-9.58e-07 ***	-1.53e-06, -3.88e-07
Smoking	No		
	Yes	-1.33	-5.50, 2.85
Alcohol consumption	No	0	0.00, 0.00
	Yes	1.6	-1.20, 4.39
Physical activity (METs/min)		-0.00011	-0.00042 0.0002
Baseline BMI (kg/m^2)		0.37*	0.04, 0.71
Difference in BMI (kg/m^2)		-2.28**	-3.87, -0.70
Antihypertensive medication	No		
	Yes	-1.09	-4.11, 1.94
Baseline heart rate (beats/minute)		0.12	-0.27, 0.50
Difference in heart rate		-0.24	-0.67, 0.20
Baseline systolic blood pressure		0.62***	0.42, 0.82
Treatment allocation	Control		
	Intervention	-6.18***	-7.93, -4.42

Note: †, Model included a dichotomous independent variable representing belonging to the intervention group ("1") or control group ("0") and trial centre as a second-level variable; Adjusted for age, gender, marital status, ethnicity, education, occupation, income, smoking, alcohol consumption, physical activity, body mass index (BMI), change in resting heart rate, and baseline systolic blood pressure;
§, 95% confidence interval for B;
*, p <0.05; **, p <0.01; ***, p<0.001

Table 2. Intervention effects on diastolic blood pressure

Characteristic		Model 4†	
		**B**	**CI§**
Age (years)		0.11	-0.03, 0.24
Gender	Male		
	Female	0.71	-1.17, 2.60
Ethnicity	Brahman		
	Chhetri	1.33*	0.28, 2.37
	Janajati	-0.73	-3.50, 2.04
	Others	2.61	-0.55, 5.77
Marital status	Married		
	Others	-1.64*	-2.96, -0.33
Education		0.02	-0.16, 0.16
Occupation	Job		
	self-employed	-3.02***	-4.34, -1.71
	Homemaker	-2.37*	-4.24, -0.49
	Others	1.04	-1.53, 3.62
Household income (Nepali Rupees)		-7.43e-07 ***	-1.18e-06 -3.08e-07
Smoking	No		
	Yes	-1.23	-3.70, 1.24
Alcohol consumption	No		
	Yes	1.24	-0.45, 2.93
Physical activity (METs/min)		0.00017	-0.00034 0.00067
Basline BMI (kg/m^2)		0.08	-0.19, 0.35
Difference in BMI (kg/m^2)		-2.30***	-3.57, -1.03
Antihypertensive medication	No		
	Yes	-1.13	-3.26, 0.99
Baseline heart rate (beats/minute)		0.15	-0.04, 0.34
Difference in heart rate (beats/minute)		-0.08	-0.42, 0.25
Baseline diastolic blood pressure		0.41**	0.16, 0.67
Treatment allocation	Control		
	Intervention	-2.65*	-4.84, -0.46

Note: †, Model included a dichotomous independent variable representing belonging to the intervention group (“1”) or control group (“0”) and trial centre as a second-level variable; Adjusted for age, gender, marital status, ethnicity, education, occupation, income, smoking, alcohol consumption, physical activity, body mass index (BMI), change in resting heart rate, and baseline systolic blood pressure;
§, 95% confidence interval for B;
*, p <0.05; **, p <0.01; ***, p<0.001
