# Supplementary material for: Effects of a health worker-led 3-month yoga intervention on blood pressure of hypertensive patients: a randomised controlled multicentre trial in the primary care setting
Source: BMC Public Health. 2021 Mar 20;21:550. doi: 10.1186/s12889-021-10528-y (PMC7981931; doi:10.1186/s12889-021-10528-y)
Supplement: Supplementary file 9 — Additional file 9. Marginal plots. [file 12889_2021_10528_MOESM9_ESM.pdf]

Additional file 9. Marginal plots for antihypertensive medication, gender, smoking, and alcohol consumption

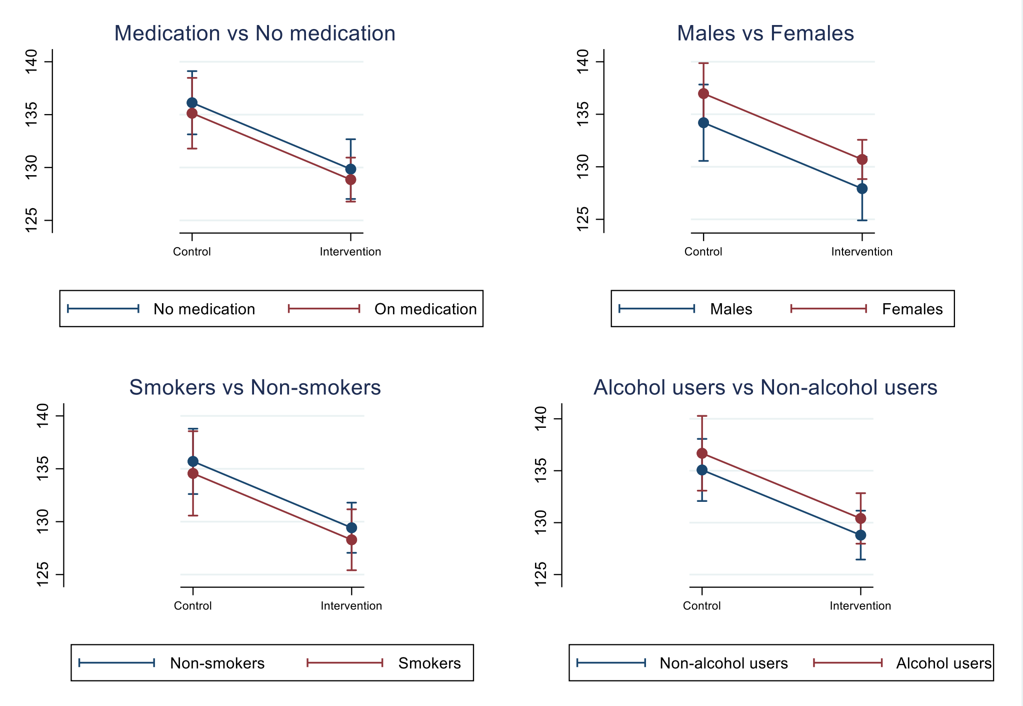

Note: y axis= Systolic blood pressure (mmHg)
