# Supplementary material for: Effects of a health worker-led 3-month yoga intervention on blood pressure of hypertensive patients: a randomised controlled multicentre trial in the primary care setting
Source: BMC Public Health. 2021 Mar 20;21:550. doi: 10.1186/s12889-021-10528-y (PMC7981931; doi:10.1186/s12889-021-10528-y)
Supplement: Supplementary file 10 — Additional file 10. Findings from fidelity assessment. [file 12889_2021_10528_MOESM10_ESM.pdf]

Additional file 10. Findings from fidelity assessment

	**Question**	**Response**	**n(%)**
1.	Have you attended the yoga training conducted by a District Ayurveda Health Centre?	Yes	12 (100.0%)
		No	0 (0.0%)
2.	For how many days did you attend the training?	5 days	12 (100.0%)
		< 5 days	0 (0.0%)
3.	Was the training useful for you to learn yoga? Please rate its usefulness on the scale from 0 to 5, where 0 stands for “not at all useful” and 5 stands for “extremely useful”.	Average score (mean)	4.3
		Standard deviation	0.9
4.	Were you satisfied with the yoga training provided at the health centre? Please rate your satisfaction level on the scale from 0 to 5, where 0 stands for “not satisfied” and 5 stands for “extremely satisfied”.	Average score (mean)	4.6
		Standard deviation	0.5
5.	Did you practise yoga at home as part of the YoH trial, as recommended?	Yes	12 (100.0%)
		No	0 (0.0%)
6.	How often did you practise yoga at home in this period?	Regularly	12 (100.0%)
		Sometimes	0 (0.0%)
		Rarely	0 (0.0%)
		Never	0 (0.0%)
		I don’t remember	0 (0.0%)
7.	During your home-based yoga sessions, did you follow the structure recommended by the health workers?	Yes	12 (100.0%)
		No	0 (0.0%)
8.	Which yoga exercises did you practise regularly?	“Omkar”	12 (100.0%)
		Warm-up exercises	12 (100.0%)
		Yogic abdominal awareness	12 (100.0%)
		Lateral Arc Pose	12 (100.0%)
		Twist Pose	12 (100.0%)
		Left nostril breathing	12 (100.0%)
		Cooling breathing	12 (100.0%)
		Alternate nostril breathing	12 (100.0%)
		Humming bee breathing	12 (100.0%)
		Yogic sleep	12 (100.0%)
		Other (please list)------	0 (0.0%)
